# Data analyses of honokiol-induced autophagy of human glioma cells *in vitro* and *in vivo*

**DOI:** 10.1016/j.dib.2016.09.045

**Published:** 2016-10-08

**Authors:** Gong-Jhe Wu, Chien-Ju Lin, Yung-Wei Lin, Ruei-Ming Chen

**Affiliations:** a Department of Anesthesiology, Shin Kong Wu Ho-Su Memorial Hospital, Taipei, Taiwan; b Anesthetics and Toxicology Research Center and Department of Anesthesiology, Taipei Medical University Hospital, Taipei, Taiwan; c Graduate Institute of Medical Sciences and Comprehensive Cancer Center, Taipei Medical University, Taipei, Taiwan; d Brain Disease Research Center, Taipei Medical University Wan-Fang Hospital, Taipei, Taiwan

**Keywords:** Autophagy, Human glioma cells, *in vitro*, *in vivo*

## Abstract

This article contains raw and processed data related to a research, “Honokiol induces autophagic cell death in malignant glioma through reactive oxygen species-mediated regulation of the p53/PI3K/Akt/mTOR signaling pathway” (C.J. Lin, T.L. Chen, Y.Y. Tseng, G.J. Wu, M.H. Hsieh, Y.W. Lin, R.M. Chen, 2016) [1]. Data were obtained by immunoblotting analyses of light chain 3 (LC3)-II, beclin-1, Akt, and mTOR in human glioma U87 MG cells and mouse glioma tissues treated with honokiol, an active constituent extracted from the bark of *Magnolia officinalis, “*Honokiol induces autophagy of neuroblastoma cells through activating the PI3K/Akt/mTOR and endoplasmic reticular stress/ERK1/2 signaling pathways and suppressing cell migration” (P.S. Yeh, W. Wang, Y.A. Chang, C.J. Lin, J.J. Wang, R.M. Chen, 2016) [2]. The processed data show the effects of honokiol on induction of autophagy in human glioma U87 MG cells by analyzing levels of LC3-II, p62, and bectin-1, “Honokiol-induced apoptosis and autophagy in glioblastoma multiforme cells” (K.H. Chang, M.D Yan, C.J. Yao, P.C. Lin, G.M. Lai, 2013) [3]. In addition, chloroquine, a lysosomal inhibitor, was administered to the cells to further confirm honokiol-induced cell autophagy. Sequentially, mice with gliomas were created and treated with honokiol. Amounts of phosphorylated and non-phosphorylated Akt and mTOR in glioma tissues were analyzed to determine the possible mechanisms of honokiol-induced autophagy.

**Specifications Table**

**Table:** 

Subject area	*Biology*
More specific subject area	*Biological medicine*
Type of data	*Images, figures*
How data was acquired	*Raw data by immunoblotting assays*
	*Quantified data by statistical analyses*
Data format	*Raw, quantified*
Experimental factors	*Human glioma cells were cultured and treated with honokiol or chloroquine. Mice with glioma were created and treated with honokiol.*
Experimental features	*Proteins from human glioma cells and mouse glioma tissues were isolated and separated using SDS-PAGE. Immunoblotting analyses of autophagy-related proteins were carried out and protein bands were quantified and statistically analyzed.*
Data source location	*Graduate Institute of Medical Sciences, Taipei Medical University, Taipei, Taiwan; Brain Disease Research Center, Taipei Medical University Wan-Fang Hospital, Taipei, Taiwan*
Data accessibility	*All data are with this article.*

**Value of the data**

• These data are acquired from *in vitro* and *in vivo* models of human glioma and these models are helpful for drug discovery for brain tumors.
• The present data provide several lines of evidence explaining if honokiol induced autophagic insults to human glioma cells.
• These data validate the possible mechanisms illustrating how honokiol induced autophagy of human glioma cells.

## 1 Data

**Fig. 1:**
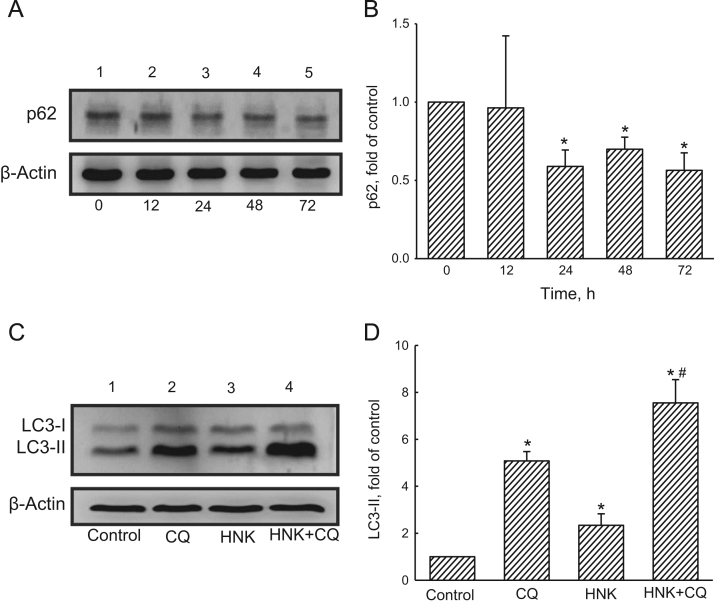
Immunoblotting analyses of an autophagic flux in human glioma cells. Human glioma U87 MG cells were exposed to 40 µM honokiol for 12, 24, 48, and 72 h. Levels of p62 and LC3 I/II were immunodetected (A and C, top panels). β-Actin was detected as the internal standard (bottom panels). These protein bands were quantified and statistically analyzed (B and D). Each value represents the mean±SEM from three independent experiments. ^⁎^ Values significantly differed from the control group, *p*<0.05.

**Fig. 2:**
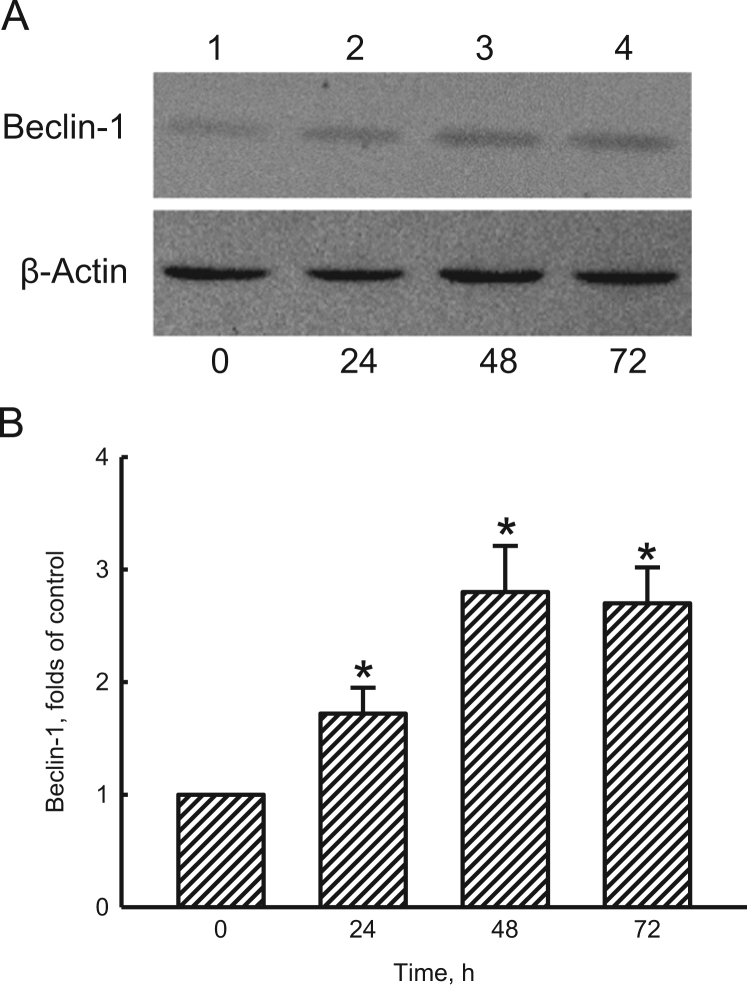
Protein assay of beclin-1, an upstream modulator of autophagy, in human glioma cells. Human glioma U87 MG cells were exposed to 40 µM honokiol for 24, 48, and 72 h. Levels of Beclin-1 were immunodetected (A, top panel). β-Actin was detected as the internal standard (bottom panel). These protein bands were quantified and statistically analyzed (B). Each value represents the mean±SEM from three independent experiments. ^⁎^ Values significantly differed from the control group, *p*<0.05.

**Fig. 3:**
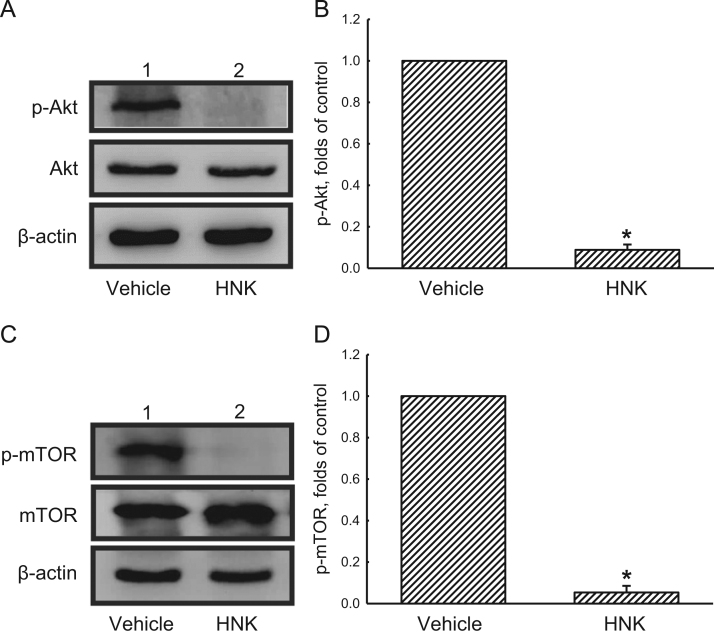
Immunoblotting analyses of phosphorylated (p) and non-phosphorylated Akt and mTOR in mouse glioma tissues. Mice with an intracranial glioma and administration of honokiol (HNK) were described in "Experimental design, materials and methods". After being sacrificed, mice brains were removed for immunoblotting analyses of Akt, p-Akt, mTOR, and p-mTOR (A and C). Levels of β-actin were analyzed as the internal control (B and D). Each value represents the mean±SEM from three independent experiments. ^⁎^ Values significantly differed from the control group, *p*<0.05.

To elucidate the honokiol (HNK)-induced autophagic flux, human glioma cells were exposed to HNK, and levels of p62, a biomarker of autophagy, were analyzed (Fig. 1A and B). Chloroquine (CQ), a lysosomal inhibitor, was added into human glioma cells to confirm the HNK-induced cell autophagy (Fig. 1C and D). In addition, amounts of beclin-1, an upstream modulator of autophagy, in human glioma cells were further analyzed (Fig. 2). An intracranial model was established to test the effects of HNK on induction of autophagy in development of glioma by analyses of phosphorylated (p) and non-phosphorylated Akt and mTOR (Fig. 3).

## 2 Experimental design, materials and methods

### 2.1 Cell culture and drug treatment

Human U87 MG cells purchased from American Type Culture Collection (Manassas, VA, USA) were seeded in minimum essential medium (MEM, Gibco-BRL Life Technologies, Grand Island, NY, USA) [3]. The MEM was supplemented with 10% fetal bovine serum, 2 mM L-glutamine, 100 IU/mL penicillin, 100 mg/mL streptomycin, 1 mM sodium pyruvate, and 1 mM nonessential amino acids. Human U87 MG cells were grown in a humidified atmosphere of 5% CO_2_ at 37 °C. Honokiol was purchased from Sigma (St. Louis, MO, USA) and dissolved in dimethyl sulfoxide. Human U87 MG Cells were exposed to honokiol for different intervals. For the inhibition assays, the cells were pretreated with chloroquine, a lysosomal inhibitor, for 1 h and then treated with honokiol.

### 2.2 Animal orthotopic brain tumor model and drug treatment

An intracranial glioma model was created as described previously [1]. All procedures in this study were approved by the Institutional Animal Care and Use Committee of Taipei Medical University (Taipei, Taiwan). Briefly, female nude mice (BALB/c *nu*/*nu*) were purchased from the National Laboratory Animal Center, Taipei, Taiwan. Human U87 MG cells (2×10^5^ cells) were suspended in 3 µl phosphate-buffered saline and then stereotactically inoculated into the right frontal lobe using a syringe pump (SINGA Technology, Taipei, Taiwan). After implantation for 4 days, these glioma-bearing mice were intraperitoneally injected with 20 mg/kg honokiol or vehicle two times per week for 2 weeks. The animals were sacrificed following implantation of glioma cells for 3weeks. Brains were removed for further analyses. Gliomas were measured according to a previously described method [1].

### 2.3 Immunoblotting

Human U87 MG cells or mouse brain tissues were lysed with an ice-cold lysis buffer (25 mM HEPES, 1.5% Triton X-100, 0.1% sodium dodecylsulfate (SDS), 0.5 M NaCl, 5 mM EDTA, and 0.1 mM sodium deoxycholate) as described previously [3], [4]. Proteins were separated using SDS-polyacrylamide gel electrophoresis and then transferred onto nitrocellulose membranes. After blocking with a 5% skim milk solution, the membranes were incubated with indicated antibodies against LC3, beclin-1, phosphorylated (p)-Akt, Akt, p-mTOR, and mTOR (Cell Signaling Technology (Beverly, MA, USA) and p62 (Santa Cruz Biotechnology, Santa Cruz, CA, USA). Membranes were probed with the appropriate secondary antibodies at room temperature. Those immunoreactive proteins were detected using an enhanced chemiluminescence reagent (Western Lightning^TM^ Plus-ECL, Perkin-Elmer, Waltham, MA, USA). Levels of β-actin were immunodetected as the internal control (Sigma). The density of these protein bands were quantified using a digital analyzer [2].

### 2.4 Statistical analysis

The statistical significance of differences between the control and drug-treated groups was evaluated using Student׳s *t*-test; differences between drug-treated groups were evaluated using Duncan׳s multiple-range test. Statistical analyses between groups over time were carried out by a two-way analysis of variance. A *p* value of <0.05 was considered statistically significant.
